# Chest palpitations in a teenager as an unusual presentation of Lyme disease: case report

**DOI:** 10.1186/s12879-020-05438-0

**Published:** 2020-10-07

**Authors:** Faith Myers, Pooja E. Mishra, Daniel Cortez, Mark R. Schleiss

**Affiliations:** 1 Department of Pediatrics, Masonic Children’s Hospital, Pediatric Medical Education, 2450 Riverside Ave, Minneapolis, MN 55454 USA; 2 Division of Pediatric Cardiology, University of Minnesota Medical School, 2450 Riverside Ave, Minneapolis, MN 55454 USA; 3 Division of Pediatric Infectious Diseases and Immunology, University of Minnesota Medical School, 2001 6th Street SE, Minneapolis, MN 55455 USA

**Keywords:** *Borrelia burdorferi*, Lyme disease, Lyme carditis, Atrioventricular block (AVB)

## Abstract

**Background:**

The incidence of Lyme disease (LD) in North America has increased substantially in the past two decades. Concomitant with the increased incidence of infection has been an enhancement in the recognition of LD complications. Here, we report a case of Lyme carditis complicated by heart block in a pediatric patient admitted to our children’s hospital. What is unique about this case is that the complaint of chest palpitations is an infrequent presentation of LD, and what it adds to the scientific literature is an improved understanding of LD in the pediatric population.

**Case presentation:**

The patient was a 16-year-old male who presented with the main concerns of acute onset of palpitations and chest pain. An important clinical finding was *Erythema migrans* (EM) on physical exam. The primary diagnoses were LD with associated Lyme carditis, based on the finding of 1st degree atrioventricular heart block (AVB) and positive IgM and IgG antibodies to *Borrelia burgdorferi*. Interventions included echocardiography, electrocardiography (EKG), and intravenous antibiotics. The hospital course was further remarkable for transition to 2nd degree heart block and transient episodes of complete heart block. A normal sinus rhythm and PR interval were restored after antibiotic therapy and the primary outcome was that of an uneventful recovery.

**Conclusions:**

Lyme carditis occurs in < 5% of LD cases, but the “take-away” lesson of this case is that carditis can be the presenting manifestation of *B. burgdorferi* infection in pediatric patients. Any patient with suspected Lyme carditis manifesting cardiac symptoms such as syncope, chest pain, or EKG changes should be admitted for parenteral antibiotic therapy and cardiac monitoring. The most common manifestation of Lyme carditis is AVB. AVB may manifest as first-degree block, or may present as high-grade second or third-degree block. Other manifestations of Lyme carditis may include myopericarditis, left ventricular dysfunction, and cardiomegaly. Resolution of carditis is typically achieved through antibiotic administration, although pacemaker placement should be considered if the PR interval fails to normalize or if higher degrees of heart block, with accompanying symptoms, are encountered. With the rising incidence of LD, providers must maintain a high level of suspicion in order to promptly diagnose and treat Lyme carditis.

## Background

Lyme disease is the most common tick-borne illness in North America. Climate change is one of the factors that appears to have increased the prevalence of Lyme disease, due to increases in the population of tick vectors [1, 2]. The upper Midwestern United States, in particular Minnesota and Wisconsin, have seen substantial increases in LD and other tick-born infections in the past two decades transmitted by the deer tick, *Ixodes scapularis* [3]. Concurrent with the increased incidence of LD, an increase in cases of Lyme carditis has also been reported. It is known that cardiac presentations of Lyme carditis may include myocarditis [4], sick sinus syndrome [5], and endocarditis [6, 7], but the most common manifestation is AV nodal block [7]. We describe in this case report a 16-year-old male who presented to our hospital with the chief complaint of palpitations and chest pain and was discovered to have first degree heart block. His admission history and physical examination strongly suggested LD, which was confirmed by laboratory evaluation.

This unusual but important presentation of pediatric LD was noted against the backdrop of the ongoing increase in LD prevalence in Minnesota. What makes this case report unique is that the patient’s main concerns were chest pain and palpitations, representing unusual presenting manifestations of LD. We review this presentation and discuss it in the context of the medical literature, citing relevant references. This case adds to the medical literature, both by alerting clinicians to the fact that LD is an emerging infectious disease and by demonstrating that an unusual manifestation of infection, such as carditis, can herald the onset of LD. LD should be considered in the differential diagnosis of carditis, with or without conduction system disturbances, in the pediatric population.

## Case presentation

**Fig. 1:**
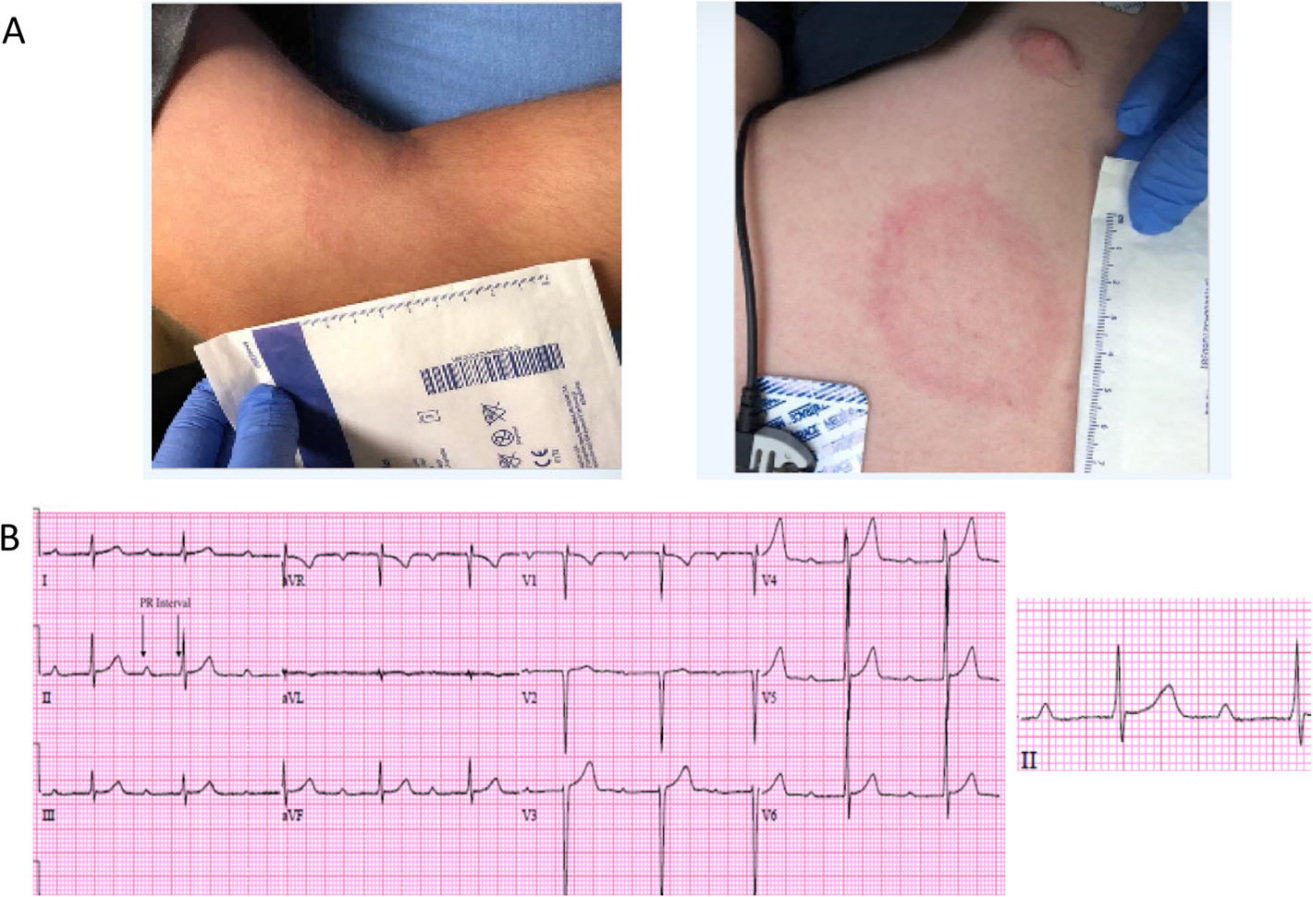
**a** Multiple EM lesions as an unexpected finding on admission. The patient in this case presented with a chief complaint of chest palpitations, but EM lesions were noted as an incidental finding at the time of initial physical exam upon hospital admission, involving the leg (left panel), and shoulder (right panel). Lyme ELISA and IgM western blot confirmed the diagnosis of *Borrelia burgdorferi* infection. **b** First degree AVB in pediatric Lyme carditis. The patient was noted to have first degree AVB upon initial evaluation (left panel). PR interval after hospitalization, 396 msec (right panel). PR interval remained prolonged for the first 10 days of IV antibiotics, and transient episodes of second and third degree AVB were noted, but after day 10 the patient was in a sustained sinus rhythm with normal PR interval and was transitioned to oral antibiotics

A 16-year-old male presented with the primary concerns and symptoms of the acute onset of palpitations and generalized chest pain. Although not noted by the patient and not a component of his chief complaint, during his initial physical examination, he was also noted to have a rash (Fig. 1a) compatible with EM. Cardiac exam revealed an irregular rhythm. No other concerns were noted on exam. The patient did not recall having been bitten by a tick. The medical, family, and psychosocial history, including relevant genetic information, was reviewed and found to be noncontributory. No prior interventions had been undertaken. An EKG demonstrated 1st degree AV block with a PR interval of 384 msec (normal range, 120–200 msec). Laboratory evaluation on the day of admission revealed a normal basic metabolic panel (BMP) and magnesium level. His troponin I (extra-sensitive) level was 0.042 μg/L with a reference range of 0.000–0.045 μg/L (M Health Fairview Ridges Hospital Lab). His complete blood count with differential was within normal limits. The patient’s C-reactive protein level on the day of admission was 34.3 mg/L (reference range, 0.00–8.0 mg/L). Group A *Streptococcus* was not detected via PCR of a pharyngeal swab. Anti-DNase B and antistreptolysin O antibodies were not detected.

Diagnostic methods included an echocardiogram on hospital day one that revealed normal intracardiac connections, normal motion of the tricuspid, mitral, pulmonary and aortic valves, and no pericardial effusion. There was normal left ventricular size and left ventricular systolic function. The calculated left ventricular ejection fraction from the four-chamber view was 53%. An echocardiogram was repeated on hospital day five and revealed normal valves and motion, no pericardial effusion, and a calculated biplane left ventricular ejection fraction of 70%. Laboratory studies revealed detectable *Borrelia burgdorferi* antibodies by ELISA assay, with an index value of 7.91 (reference range 0.00–0.89). *B. burgdorferi* IgM was detected via western blot assay (bands present: 41, 39, 23 kDa). Differential diagnostic considerations included other vector borne diseases, and a post-streptococcal inflammatory process. However, streptococcal studies were negative, and laboratory evaluation did not detect *Anaplasma phagocytophilum*, *Ehrlichia chaffeensis*, or *Ehrlichia muris*-like DNA by PCR (ARUP laboratories, SLC, Utah, USA). *Anaplasma phagocytophilum, Babesia microti* and *Ehrlichia* antibody studies were all negative. In addition, no *Babesia, Anaplasma phagocytophilum* or *Ehrlichia* species were found on a parasite (Giemsa) stain of the blood. Thus, the final diagnosis was LD, with associated Lyme carditis.

Diagnostic challenges included an evolving rhythm profile during the hospitalization. A stress test was performed on hospital day nine following transient episodes of complete heart block. The patient exercised for 8 min and 3 s, achieving a heart rate of 187 bpm in sinus rhythm. He stopped due to his legs feeling tired. He achieved 91.75% of his expected maximum sinus rate. There were no arrhythmias or ST-T changes. A BMP rechecked on hospital day eight remained normal. First degree AVB continued (Fig. 1b). During the first week of hospitalization, he was noted to have sinus pauses and possible 2:1 heart block on telemetry, and a brief episode of third degree AVB.

**Fig. 2:**
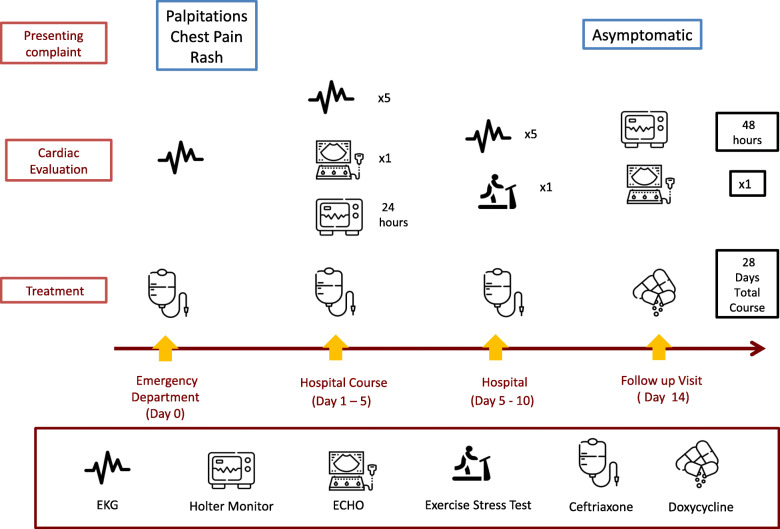
Timeline of clinical course schematically demonstrating workup and treatment of Lyme carditis. Figure icons from Flaticon (https://www.flaticon.com/home)

For therapeutic intervention, the patient was treated with IV ceftriaxone for 10 days with continuous cardiac monitoring until sustained stabilization of the PR interval at < 200 msec was demonstrated. His PR interval normalized to < 176 msec after 10 days of parenteral antibiotic treatment. He was transitioned to oral doxycycline (see timeline, Fig. 2) and discharged home to complete a 28-day course of antibiotics.

Upon follow-up, extended ambulatory cardiac monitoring demonstrated resolution of the previous EKG changes. Clinician- and patient-assessed outcomes were both assessed as highly favorable. The important follow-up diagnostic test results focused on EKG, which normalized. Intervention adherence and tolerability, as assessed by careful history, was exemplary. There were no adverse or unanticipated events. The patient shared his perspective on the treatment he received and commented that he was “doing well”. He denied palpitations, chest pain, syncope or presyncope and was overall “doing well without concern”.

## Discussion and conclusion

The prevalence of LD has steadily increased in the United States over the past two decades, particularly in the upper Midwest. The increased prevalence appears to be driven by climate change, with resultant expansion of the tick habitat and an increased number of tick vectors [1, 3]. With the increase in total LD cases, a concomitant 2.4-fold increase in Lyme carditis in children was noted in a study comparing total cases in 2007 to cases in 2013 [8]. Lyme carditis in children is rare, with an overall incidence of < 5% [9]. The most common manifestations of Lyme carditis are heart block (first-degree block, or high-grade second or third-degree AVB) and, less frequently, myopericarditis, left ventricular dysfunction, and cardiomegaly. The spectrum of Lyme carditis is broad, and ranges from asymptomatic or mildly symptomatic first-degree heart block to fulminant myocarditis. Complete heart block [10] and, rarely, sudden death [11] have been described with Lyme carditis. A case-control study of 207 children with early disseminated LD [12] demonstrated that 33 (16%) had carditis, and 14 (42%) of this sub-group had advanced heart block (including 9 [27%] children with complete heart block). Four (12%) children in this group had depressed ventricular systolic function, and 3 (9%) of these children required temporary pacing. The prevalence of Lyme carditis in children may be underestimated. In a study of children with definitive LD without cardiac symptoms, 29% of children had an abnormal EKG (most commonly first-degree heart block). Although this was a small case series and requires confirmation in larger studies, this report nonetheless demonstrated the importance of maintaining an appropriate index of clinical suspicion for investigating potential cardiac manifestations in children presenting with evidence of LD [13].

The pathogenesis of Lyme carditis is uncertain. The injury appears to be related to a pathologic immune response to infection. In a post-mortem study of Lyme carditis, cardiac myocyte necrosis was minimal, but lymphocytic infiltrates were noted, including T cells and plasma cells [14]. Spirochetes were also identified in the cardiac interstitium in association with collagen fibers, co-localized with decorin, a proteoglycan associated with collagen fibrils. *Borrelia burgdorferi* encodes several surface proteins, including decorin- and dermatan sulfate-binding adhesins. The dermatan sulfate-binding adhesin, *DbpA*, mediates colonization by the Lyme spirochete in a strain-specific manner, with genetic variations in this gene product potentially contributing to the variability of clinical manifestations associated with different strains [15]. Another genetic element of interest in the pathogenesis of Lyme carditis maps to a genetic element known as linear plasmid 17 (lp17). A mutant spirochete lacking a ~ 4.7 kb fragment of lp17 was found to be impaired in its ability to induce carditis in a murine model [16], although the precise molecular mechanisms remain to be defined.

The cornerstone of management of Lyme carditis is cardiac monitoring, supportive care, and antibiotic therapy. In some cases, pacemaker placement may be required. Steroids do not appear to shorten the time course of Lyme carditis and are not routinely recommended [17]. For situations in which the diagnosis of LD may be uncertain, a risk score algorithm, the “Suspicious Index in Lyme Carditis (SILC)” has been developed to help predict outcomes and direct management [18]. Although developed for adult patients, calculation of the SILC risk score and accompanying “COSTAR” mnemonic (constitutional symptoms; outdoor activity; sex = male; tick bite; age < 50; rash = erythema migrans) may help to identify Lyme carditis in children presenting with high-degree AVB, and ultimately, may help minimize the implantation of unnecessary permanent pacemakers (since treatment with antibiotics can lead to resolution of AVB, obviating the need for pacing). Such scoring systems may be particularly useful for patients (such as the one described in this case report) that present with signs and symptoms of carditis prior to the emergence of EM. Lyme carditis, including cases with high-degree AVB, typically resolves with antibiotic therapy. It has been suggested that clinician choices regarding duration of antibiotic therapy and route of administration may depend upon the magnitude of AVB. It is estimated that 90% of LD patients with carditis will manifest AVB [19]. High-degree AVB typically resolves within the first 10 days of antibiotic treatment, and other less severe conduction disturbances run their course within 6 weeks [19, 20]. A marker of high risk for progression to complete AVB is an initial PR interval > 300 ms at presentation. Patients with an intermediate or high SILC score in the context of high-degree AVB, particularly if EM is present and the patient lives in a Lyme-endemic region (such as Minnesota), should be treated empirically for LD, even if Lyme diagnostic studies are pending [19]. Confirmed LD with associated carditis is typically treated with IV ceftriaxone for 10 to 14 days (although therapy for up to 28 days in patients with high-grade AVB may be required), followed by oral antibiotics (options include doxycycline, amoxicillin, or cefuroxime), usually for a total antibiotic course of 14 to 21 days [19]. The decision to transition from intravenous to oral antibiotics can be informed by the rate of normalization of the PR interval. The Infectious Diseases Society of America recommends that parenteral antibiotic therapy should be continued until high-degree AVB has resolved and the P-R interval has deceased to < 300 ms, after which the patient should be transitioned to an oral therapy [21]. High-degree AVB usually resolves in a stepwise sequence, evolving from third-degree block, to Wenckebach second-degree block, to first-degree block, to normal sinus rhythm [22]. If AVB persists after treatment, a pre-discharge stress test has been recommended to assess the stability of AV conduction. In this algorithm, if 1:1 AV conduction is maintained at a heart rate of > 120, then an outpatient oral antibiotic regimen is recommended, but if conduction fails at < 90 bpm, a permanent pacemaker may be considered [23].

Strengths in our approach to this case include serological confirmation of LD in a patient with carditis and EM. This, as noted in discussion of the relevant medical literature, provides the rationale for our conclusion that this patient’s presentation represents a case of Lyme carditis. A limitation is that we did not have endomyocardial biopsy confirmation, but biopsy is rarely conducted in Lyme carditis.

In summary, what are the main “take-away” lessons learned from our case? First, this case illustrates that signs and symptoms of cardiac disease (palpitations, chest pain) may be the chief complaint of patients with LD in pediatric practice. Although the discovery of EM on initial physical examination rapidly sharpened our focus regarding the possibility of LD in our patient, clinicians should be mindful that carditis may be the sole presenting manifestation of LD in some cases. Secondly, the use of a SILC risk score may be useful in gauging the risk of Lyme carditis, particularly in patients from Lyme-endemic regions. If the presentation is compatible with Lyme carditis, empirical intravenous antibiotics, typically ceftriaxone, should be administered pending the results of diagnostic studies for *Borrelia burdorferii*. Third, it is worth emphasizing that Lyme carditis rarely requires pacemaker placement. Symptoms and electrocardiographic abnormalities typically both respond readily to antibiotic therapy. With the escalating prevalence of LD occurring in the context of climate change, clinicians are increasingly likely to encounter Lyme carditis. Until a vaccine strategy can be refined, vector control measures and an appropriate index of suspicion on the part of clinicians will be essential in responding to this important complication of LD [24].

## Data Availability

All data and materials are available from the corresponding author.

## Abbreviations

LD: Lyme disease
AVB: Atrio-ventricular block
EM: *Erythema migrans*
SILC: Suspicious Index in Lyme Carditis
COSTAR: Constitutional symptoms; outdoor activity; sex = male; tick bite; age < 50; rash = erythema migrans
EKG: Electrocardiogram
UMN: University of Minnesota
